# BCL11A Is Oncogenic and Predicts Poor Outcomes in Natural Killer/T-Cell Lymphoma

**DOI:** 10.3389/fphar.2020.00820

**Published:** 2020-06-04

**Authors:** Hongyun Shi, Chun Li, Wei Feng, Jianjun Yue, Jingfang Song, Aizhi Peng, Hua Wang

**Affiliations:** ^1^Department of Pediatrics, The Second Affiliated Hospital of University of South China, Hengyang, China; ^2^Department of Hematological Oncology, Sun Yat-sen University Cancer Center, State Key Laboratory of Oncology in South China, Collaborative Innovation Center for Cancer Medicine, Guangzhou, China

**Keywords:** B‐cell chronic lymphocytic leukemia/lymphoma 11A, natural killer/T-cell lymphoma, gene expression, oncogenic, prognosis

## Abstract

The current treatment for natural killer/T-cell lymphoma (NKTL) among advanced/relapsed patients is unsatisfying, thereby highlighting the need for novel therapeutic targets. B‐cell chronic lymphocytic leukemia/lymphoma 11 A (BCL11A), as a transcription factor, is oncogenic in several neoplasms. However, its function in NKTL remains unclear. Quantitative real-time polymerase chain reaction and Western blot analysis were used to measure the BCL11A expression levels among NKTL patients and in NKTL cell lines. Natural killer (NK) cells from healthy subjects were used as negative control. Transient transfection with small interfering RNA was used to knockdown the expression in NKTL cell lines. Samples and clinical histories were collected from 343 NKTL patients (divided into test and validation groups) to evaluate the clinical value of BCL11A expression level. The BCL11A expression was upregu\lated among NKTL patients and in NKTL cell lines. Reduced cell proliferation and increased apoptosis were observed after silencing BCL11A in NKTL cell lines. BCL11A expression level was correlated with RUNX3, c-MYC, and P53 in NKTL. Notably, a high BCL11A expression was correlated with unfavorable clinical characteristics and predicted poor outcomes in NKTL. In conclusion, BCL11A was overexpressed in NKTL, while its upregulation promoted tumor development. Therefore, BCL11A expression level may be a promising prognostic biomarker for NKTL.

## Introduction

Natural killer/T-cell lymphoma (NKTL) is an aggressive, rare disease that is more frequently observed in Asia and South America than in North America and Europe (Tse and Kwong, 2016). NKTL is a neoplastic disease characterized by T and natural killer (NK) cells with Epstein–Barr virus (EBV) infection (Tse and Kwong, 2017). Much progress has been reported in the development of NKTL treatment strategies. The first-line treatment for early-stage NKTL is combined chemotherapy and radiotherapy (RT) (sequentially or concurrently) (Bi et al., 2013; Wang et al., 2013). However, an optimal therapy for patients with late-stage or relapsed NKTL has not yet been formulated. Moreover, advanced/relapsed NKTL patients continue to demonstrate poor outcomes. Therefore, novel therapeutic targets are urgently needed.

B‐cell chronic lymphocytic leukemia/lymphoma 11 a (BCL11A) is a C2H2-type zinc-finger transcription factor that plays an important role in pre–B-cell development, thymocyte maturation, and globin switching (Liu et al., 2003; Sankaran et al., 2009). BCL11A is oncogenic and aberrantly expressed in several hematopoietic malignancies, including leukemia, B-cell non-Hodgkin lymphoma (B-NHL), and Hodgkin’s disease (HD) (Satterwhite et al., 2001; Yu et al., 2012; Xutao et al., 2018). BCL11A has been recently reported to function as an oncogene in several solid neoplasms, such as those of breast, lungs, prostate, and larynx (Khaled et al., 2015; Zhang et al., 2016; Lazarus et al., 2018). However, the role of BCL11A in NKTL remains unknown. Selvarajan et al. (2017) reported that RUNX3 plays an important role in NKTL as an oncogene and is transcriptionally regulated by MYC. Another study (Ng et al., 2011) demonstrated that P53 and c-MYC are overexpressed and may participate in activated oncogenic pathways in NKTL. Therefore, investigating the correlation of BCL11A with RUNX3, c-MYC, and P53 expression may help reveal the function of BCL11A in NKTL.

Accordingly, this study examined whether BCL11A is overexpressed in NKTL and explored its role in NKTL. The correlation of BCL11A with RUNX3, c-MYC, and P53 expression was also examined along with the clinical value of BCL11A expression level in NKTL.

## Materials and Methods

### Cell Culture and NK Cell Isolation

Several NKTL cell lines, including SNK-1, NK-YS, HANK-1, and KHYG-1, were incubated at 37°C in a humidified atmosphere of 5% CO_2_. **Table S1** in the supplement file shows the culture conditions in detail. The normal NK cells extracted from healthy subjects were used as negative control. Whole blood samples were obtained from healthy donors from the Blood Donation Center. Peripheral blood mononuclear cells (PBMCs) were separated by using Histopaque^®^-1077 (Sigma, Germany) *via* density gradient centrifugation. Highly pure untouched normal NK cells were isolated by using an indirect magnetic labeling system to deplete magnetically labeled cells from human PBMCs (BD Biosciences, USA). The purity of the isolated NK cells ranged from 90% to 99% according to flow cytometry.

### Immunohistochemistry

Sixteen randomly selected NKTL patient samples were stained to assess the immunohistochemical expressions of BCL11A, RUNX3, c-MYC, and P53 proteins. Anti-human primary antibodies to RUNX3, BCL11A, MYC, and P53 (ab92336, ab242406, ab32072 and ab32389, Abcam, UK) were used in Immunohistochemistry (IHC) (1:500). HRP-conjugated secondary antibodies (1:250, Thermo Scientific) were used in IHC secondary staining. Antigen retrieval was performed at 120°C for 5 min by using a pressure cooker followed by an overnight incubation at 4°C. The appropriate positive tissue controls were used. The expression levels of BCL11A and RUNX3 were scored as percentages of the total tumor cell population per 1 mm core diameter (400×). The percentages of BCL11A and RUNX3 cells in three representative high-power fields of individual samples were analyzed. The positive expression for BCL11A and RUNX3 was defined as positive nuclear expression in at least 50% of the tumor cell population.

Samples from a retrospective cohort of 227 NKTL patients were stained to assess the immunohistochemical expression level of BCL11A. The intensities of BCL11A staining were scored from 0 to 4, with 0–1, 1–2, 2–3, and 3–4 indicating no, weak, medium, and strong staining, respectively. Individual samples were blindly evaluated by at least 2 pathologists, and scores of ≥ 2 and < 2 indicated high and low expressions, respectively.

### BCL11A Knockdown

Cell lines SNK-1, NK-YS, HANK-1, and KHYG-1 were transiently transfected with small interfering RNA (siRNA) (Santa Cruz Biotechnology) targeting BCL11A according to the manufacturer’s instructions. A total of 1 × 10^6^ cells were seeded for the transient transfection. A nontargeting siRNA was used as control, and lipo2000 was used to assess the basal expression level of BCL11A. Protein and messenger RNA (mRNA) were extracted from the treated cell lines to evaluate the expression levels. **Table S2** in the supplement file presents the siRNA sequences.

### Apoptosis

Cell lines SNK-1, NK-YS, HANK-1, and KHYG-1, which were transfected with BCL11A-targeting siRNA, nontargeting siRNA, and lipo2000, were examined to evaluate the apoptotic cell death rate *via* flow cytometric analysis. A total of 1 × 10^6^ cells were seeded for the transient transfection. Apoptotic cell death analyses were carried out by using Annexin-V-APC and propidium iodide detection systems. The staining of apoptotic cells was assayed by using the Annexin-V Apoptosis Detection Kit (BD Bioscience, USA) according to the manufacturer’s instructions, and the analysis was performed on a BD LSR II (BD Bioscience, USA) flow cytometer by using the BD FACSDiva™ software.

### Cell Proliferation

Cell lines SNK-1, NK-YS, HANK-1, and KHYG-1, which were transfected with BCL11A-targeting siRNA, nontargeting siRNA, and lipo2000, were examined to evaluate the cell proliferation rate by using the BrdU Cell Proliferation Assay Kit (EMD Chemicals, Gibbstown, NJ) according to the manufacturer’s instructions. A total of 1 × 10^6^ cells were seeded for the transient transfection. Flow cytometric analysis was then performed to measure the cell proliferation rate.

### RNA Extraction and Real-Time Quantitative PCR Analysis

Cell lines SNK-1, NK-YS, HANK-1, and KHYG-1, which were transfected with BCL11A-targeting siRNA, nontargeting siRNA, and lipo2000, were examined to evaluate the BCL11A mRNA expression level. A total of 1 × 10^6^ cells were seeded for the transient transfection. The total RNA was prepared by using the miRNeasy Mini Kit (Thermo Fisher Scientific, USA) protocol including DNaseI treatment. A reverse transcription reaction was carried out by using the High-Capacity cDNA Reverse Transcription Kit System (Promega, USA). The real-time fluorescence monitoring of the PCR products was assayed with Taqman Gene Expression Master Mix (Promega, USA) and gene-specific Taqman probes by using the BioRad IQTM5 Multicolor Real-Time PCR Detection System (BioRad, USA). The relative mRNA levels were calculated using the ΔΔCT method by comparing the amount of endogenous GAPDH in the same sample. **Table S2** in the supplement file presents the sequence of primers used for qPCR.

### Western Blot Analysis

Cell lines SNK-1, NK-YS, HANK-1, and KHYG-1, which were transfected with BCL11A-targeting siRNA, nontargeting siRNA, and lipo2000, were examined to evaluate the expression levels of BCL11A, RUNX3, MYC, and P53. A total of 1 × 10^6^ cells were seeded for the transient transfection. The treated cell pellets were suspended in a lysis buffer with a cocktail of protease inhibitors (Thermo Fisher Scientific, USA). Protein detection *via* Western blot was performed *via* an electrophoretic transfer of equal amounts of SDS-PAGE-separated proteins to polyvinyl difluoride membranes (Thermo Fisher Scientific, USA), incubated with the respective primary RUNX3, BCL11A, MYC, P53 (ab92336, ab242406, ab32072, and ab32389, Abcam, UK), and β-actin (ab179467, Abcam, UK), and exposed to HRP-conjugated secondary antibodies (ab20272, Abcam, UK) before performing chemiluminescence detection (BioRad, USA).

Refer to Methods in (Tang et al., 2019) for more details on the Western blot analysis. The above experiments were performed at least five times, and the results were quantified and presented with a representative result.

### Clinical Data and Treatment

A total of 227 NKTL patients that were newly diagnosed in the Sun Yat-sen University Cancer Center between January 2008 and September 2015 were enrolled in this study to comprise the primary test group. In order to enhance the validity of the study, regarding the potential role of BCL11A expression level as a potential prognostic biomarker in NKTL, 116 NKTL patients were recruited from the first affiliated hospital of the Hainan Medical School during the same period to comprise an independent validation group. These patients were diagnosed according to the WHO classification of hematopoietic and lymphoid tumors, and their diagnosis was further confirmed by the positive EBV-encoded RNA results obtained *via in situ* hybridization (Ito, 2004). The primary treatment was induction chemotherapy, which was followed by consolidative radiotherapy. GELOX (gemcitabine, L-asparaginase, and oxaliplatin) and EPOCH (cyclophosphamide, doxorubicin, vincristine, prednisone, and etoposide) were identified as the two main induction regimens as previously reported (Wang et al., 2014; Falzone et al., 2018; Pokrovsky and Vinnikov, 2019). Complete remission was determined based on the response criteria for non-Hodgkin’s lymphoma (Cheson et al., 1999).

### Statistical Analysis

Two groups of RUNX3 expression levels were compared by conducting Student’s t-test, and the correlation between the BCL11A and RUNX3 expression levels was evaluated by conducting Spearman’s correlation test. Overall survival (OS) was defined as the time from diagnosis to death or last follow-up, whereas progression-free survival (PFS) was defined as the time from treatment to disease progression, death, or last follow-up. Clinical characteristics were compared by performing Mann–Whitney’s U test, chi-squared test, or Fisher’s exact test. The Kaplan–Meier method and log-rank test were used to analyze and compare the survival rates. Univariate and multivariate analyses were performed to evaluate the prognostic value of clinical characteristics, including BCL11A expression level. A two-sided P-value of <0.05 was considered statistically significant. The statistical analysis was performed by using SPSS 17.0.

## Results

### BCL11A Expression Is Upregulated in NKTL

**Figure 1:**
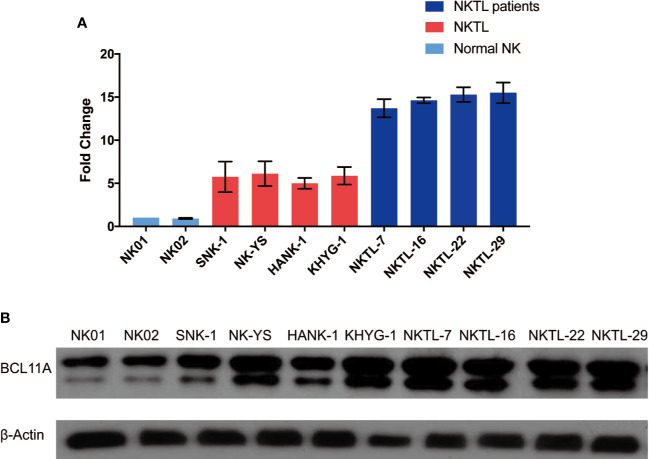
Endogenous BCL11A expression in natural killer/T-cell lymphoma (NKTL) cells (SNK-1, NK-YS, HANK-1, and KHYG-1), NKTL patient samples and normal NK cells (NK01-NK02). **(A)** messenger RNA (mRNA) expression profiles of BCL11A in NKTL cells, normal NK cells, and NKTL patient samples. cDNA was converted from total RNA of normal NK, NKTL cells, and NKTL patient samples, subsequently assayed by quantitative real-time PCR (RT-PCR). BCL11A mRNA was upregulated in NKTL cells and NKTL patient samples compared to normal NK cells. **(B)** Protein profiles of BCL11A in NKTL cells, NKTL patient samples and normal NK cells. Cell lysates were probed with BCL11A (5G4) antibody. There was overexpression of BCL11A protein in NKTL cells and NKTL patient samples compared to normal NK cells.

**Figure 2:**
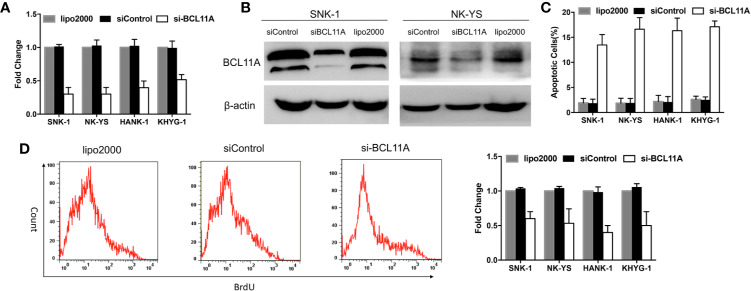
BCL11A inhibition in natural killer/T-cell lymphoma (NKTL) cells. BCL11A silenced NKTL cells (siBCL11A) showed deregulated cell proliferation and upregulated apoptosis. **(A)** NKTL cells were transiently transfected with lipo2000, control siRNA (siControl), and siRNA against BCL11A (si-BCL11A). A significant reduction of BCL11A mRNA after transfected with si-BCL11A was observed across all NKTL cells. **(B)** Whole-cell lysates were prepared and processed for immunoblotting, probed with anti-BCL11A antibody. β-Actin was used as a loading control. BCL11A protein expression was efficiently silenced after transfection with siBCL11A. **(C)** Transfected NKTL cells were stained with Annexin-V and propidium iodide and analyzed for the apoptosis rate by flow cytometry. BCL11A silenced NKTL cells showed an induction of apoptosis compared to cells transfected with control siRNA (siControl) and lipo2000. **(D)** Transfected NKTL cells were stained with BrdU and analyzed for the effects of si-BCL11A on the cell proliferation rate. The results show that cell proliferation was disrupted in BCL11A silenced NKTL cells (si-BCL11A).

**Figure 1** shows that the BCL11A mRNA and protein expression levels were upregulated in NKTL. The results suggest that the BCL11A mRNA expression level was upregulated in the cell lines and samples of NKTL patients relative to normal NK cells (**Figure 1A**). Similarly, a higher BCL11A protein expression level was observed in NKTL cell lines (SNK-1, NK-YS, HANK-1, and KHYG-1) than in normal NK cells (**Figure 1B**). An additional band of BCL11A protein, which may be the alternatively spliced isoform (Liu et al., 2006), was also observed in the NKTL cell lines (**Figures 1B**, **2B**, and **4B**). In sum, BCL11A mRNA and protein were overexpressed in NKTL.

### Knockdown of BCL11A Increases Apoptosis and Reduces Cell Proliferation

BCL11A-targeting siRNA was used to knockdown BCL11A expression and to determine the function of BCL11A in NKTL. The effect of BCL11A knockdown on apoptosis and cell proliferation was subsequently assessed. Silencing BCL11A in NKTL cell lines reduced the BCL11A mRNA and protein expressions (**Figures 2A, B**). The flow cytometric analysis of apoptosis shows that BCL11A inhibition increased the apoptosis levels in four NKTL cell lines (**Figure 2C**) and inhibited cell proliferation in NKTL cell lines (**Figure 2D**). In sum, BCL11A knockdown increased apoptosis and reduced cell proliferation in NKTL.

### BCL11A Expression Is Correlated With RUNX3 Expression Level

**Figure 3:**
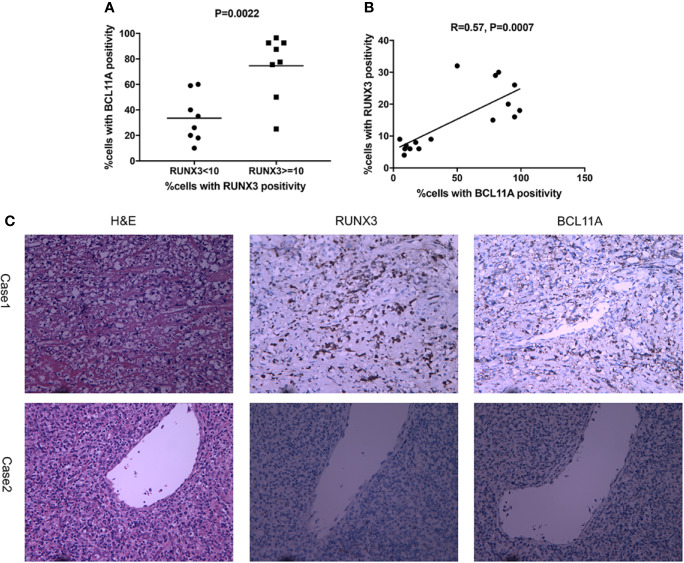
Expression of RUNX3 and BCL11A protein in 16 NKTL patient samples were examined using immunohistochemistry. **(A)** According to percentages of cells with RUNX3 positivity, cases were divided into two groups: RUNX3 < 10% and ≧̸10%. The scatter plot showed that cases with high RUNX3 protein expression (RUNX3≧̸10%) showed a significantly higher median expression of BCL11A using Student’s t-test (P=0.0022). **(B)** Spearman correlation analysis suggest moderate correlation between RUNX3 and BCL11A protein expression levels (R=0.57, P =0.0007). **(C)** Representative images of H&E-staining, immunochemistry-staining of BCL11A and RUNX3 protein in samples. BCL11A and RUNX3 expression levels were high in case 1; BCL11A and RUNX3 expression levels were low in case 2.

To further explore the role of BCL11A overexpression in NKTL, the protein expression levels of BCL11A and RUNX3 in 16 NKTL patients were measured *via* IHC. The median BCL11A expression was significantly higher in cases with a high RUNX3 protein expression (P = 0.0022; **Figure 3A**). The results of the Spearman’s correlation analysis reveal a moderate correlation between BCL11A and RUNX3 protein expressions (R= 0.57, P = 0.0007; **Figure 3B**). Representative IHC images show that both BCL11A and RUNX3 protein expression levels were high in case 1 yet are low in case 2 (**Figure 3C**), thereby suggesting a correlation between these expression levels.

**Figure 4:**
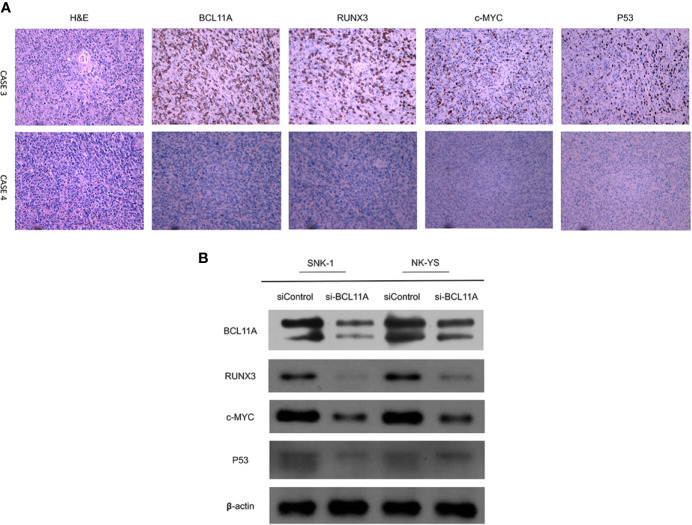
Correlation of BCL11A, RUNX3, c-MYC, and P53 protein expressing levels. **(A)** Representative images of immunohistochemical expression of BCL11A, RUNX3, c-MYC, and P53 protein in two natural killer/T-cell lymphoma (NKTL) samples. In case 3, BCL11A protein expressing level was high, along with high RUNX3, c-MYC, and P53 protein expressing levels. In contrast, case 4 with low BCL11A protein expressing level showed low RUNX3, c-MYC, and P53 protein expressing levels. **(B)** Protein expression of BCL11A, RUNX3, c-MYC, and P53 after BCL11A knockdown. After efficiently silenced BCL11A as showed in **Figure 2**, RUNX3, c-MYC, and P53 expression profiles were analyzed post 96 h after BCL11A knockdown. Silencing of BCL11A sequentially deregulated RUNX3, c-MYC, and P53 expression in NKTL cells.

The protein expression of BCL11A may also be correlated with those of RUNX3, c-MYC, and P53 (**Figure 4**). For instance, an NKTL patient with a high immunohistochemical expression of BCL11A also showed high expression levels of RUNX3, c-MYC, and P53. In another case, an NKTL patient with a low expression of BCL11A showed negative expressions of RUNX3, c-MYC, and P53 (**Figure 4A**). Immunoblotting was performed to evaluate RUNX3, c-MYC, and P53 protein expression levels in the SNK-1 and NK-YS cell lines 96 h after BCL11A knockdown. The expression levels of RUNX3, c-MYC, and P53 protein were downregulated sequentially after silencing BCL11A (**Figure 4B**). Therefore, the expression of BCL11A may be correlated with those of RUNX3, c-MYC, and P53 in NKTL cells.

### Pretreatment BCL11A Expression Is Correlated With the Survival Outcomes of NKTL Patients

**Figure 5:**
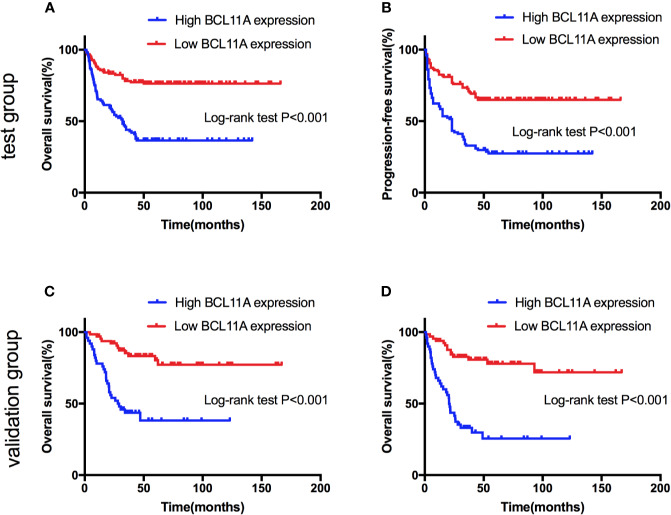
Survival curves of natural killer/T-cell lymphoma (NKTL) patients with high and low expression level of BCL11A. **(A)** Overall survival (OS) of test group of NKTL patients with high and low expression level of BCL11A. **(B)** Progression-free survival (PFS) of test group of NKTL patients with high and low expression level of BCL11A. **(C)** OS of validation group of NKTL patients with high and low expression level of BCL11A. **(D)** PFS of validation group of NKTL patients with high and low expression level of BCL11A.

The BCL11A expression levels of NKTL patients in the test (227 patients) and validation (116 patients) groups were retrospectively examined to investigate the clinical value of BCL11A in NKTL. The clinical features according to BCL11A expression level are presented in **Tables 1** (test group) and **S3** (validation group). The results from the test group suggest that patients with low and high BCL11A expression levels shared the same clinical features, except for the fact that those patients with a high BCL11A expression (24.8% versus 13.5%, P = 0.030; 42.6% versus 27.0%, P = 0.014, respectively) have late stage and high lactate-dehydrogenase (LDH) levels. After therapy, those patients with a high BCL11A expression showed a significantly lower CR rate than those with a low BCL11A expression (37.6% versus 61.9%, P < 0.001) (**Table 1**). The results from the validation group were similar to those from the test group. Late clinical stages, high LDH levels and low CR rate were correlated with high BCL11A expression levels (*P*=0.031, *P*=0.026, *P* < 0.001, respectively) (**Table S3**). **Table 2** summarizes the results of the univariate and multivariate analyses of prognostic factors for the test group. High BCL11A expression, late stage, and non-CR response were identified as independent unfavorable prognostic factors for OS and PFS. The results from validation group are consistent with those from the test group. Specifically, high BCL11A expression, late stage, and non-CR response were identified as independent adverse prognostic factors for OS and PFS (**Table S4**). In the univariate analysis of the test group, those patients with a high BCL11A expression showed significantly lower survival rates compared with those with a low BCL11A expression (5-year OS: 36.6% versus 76.3%, P < 0.001, **Figure 5A**; 5-year PFS: 27.5% versus 64.9%, P < 0.001, **Figure 5B**). As for the validation group, those patients with a high BCL11A expression showed significantly lower survival rates compared with those with a low BCL11A expression (3-year OS: 43.6% versus 83.6%, P < 0.001, **Figure 5C**; 3-year PFS: 33.1% versus 80.7%, P < 0.001, **Figure 5D**).

**Table 1:** The clinical characteristics and treatment modalities of the test group of NKTL patients.

Parameters	Total *n* (%)	BCL11A low expression *n* (%)	BCL11A high expression *n* (%)	*P* value
Overall	227(100)	126(55.5)	101(44.5)	–
Male gender	154(67.8)	85(67.5)	69(68.3)	0.891
Age >60 years	38(16.7)	24(19.0)	14(13.9)	0.298
ECOG score ≥2	42(18.5)	19(15.1)	23(22.8)	0.138
Ann Arbor stage				
I\II	185(81.5)	109(86.5)	76(75.2)	0.030
III\IV	42(18.5)	17(13.5)	25(24.8)	
B symptoms	108(47.6)	56(44.4)	52(51.5)	0.291
LDH > 245U/L	77(33.9)	34(27.0)	43(42.6)	0.014
Chemotherapy regimen				
GELOX	130(57.3)	77(61.1)	53(52.5)	0.191
EPOCH	97(42.7)	49(38.9)	48(47.5)	
Treatment response				
CR	116(51.1)	78(61.9)	38(37.6)	<0.001
Non-CR	111(48.9)	48(38.1)	63(62.4)	

NKTL, natural killer/T-cell lymphoma; BCL11A, B‐cell chronic lymphocytic leukemia/lymphoma 11 A; ECOG, Eastern Cooperative Oncology Group, LDH, lactate dehydrogenase, GELOX, gemcitabine, L-asparaginase, and oxaliplatin; EPOCH, cyclophosphamide, doxorubicin, vincristine, prednisone, and etoposide; CR, complete remission.

**Table 2:** Univariate and multivariate analyses of prognostic factors in the test group of NKTL patients.

Parameters	Overall survival			Progression-free survival		
	Univariate analysis	Multivariate analysis		Univariate analysis	Multivariate analysis	
	P value	HR (95% CI)	P value	P value	HR (95% CI)	P value
Gender						
Male vs. female	0.256			0.206		
Age						
>60 vs. ≤60years	0.493			0.312		
ECOG scores						
≥2 vs. 0-1	<0.001			<0.001		
Stage						
III\IV vs. I\II	<0.001	4.29(2.73–6.73)	<0.001	<0.001	4.09(2.66–6.30)	<0.001
B symptoms						
Yes vs. no	0.106			0.110		
LDH						
Elevated vs. normal	<0.001			<0.001		
Chemotherapy						
GELOX vs. EPOCH	0.073			0.006	1.49(1.03–2.16)	0.034
Treatment response						
Non-CR vs. CR	<0.001	1.62(1.02–2.57)	<0.001	<0.001	1.62(1.08–2.41)	0.019
BCL11A expression						
High vs. low	<0.001	2.62(1.65–4.16)	<0.001	<0.001	2.23(1.50–3.30)	<0.001

NKTL, natural killer/T-cell lymphoma; HR, hazard ratio; CI, confidence interval; ECOG, Eastern Cooperative Oncology Group; LDH, lactate dehydrogenase; GELOX, gemcitabine, L-asparaginase, and oxaliplatin; EPOCH, cyclophosphamide, doxorubicin, vincristine, prednisone, and etoposide; CR, complete remission; BCL11A, B‐cell chronic lymphocytic leukemia/lymphoma 11 A.

## Discussion

NKTL is a rare and aggressive disease with poor outcomes, especially among patients with advanced/relapsed disease. Therefore, novel therapeutic targets are urgently needed. Several reports have shown that BCL11A is oncogenic in B‐cell lymphoma, B‐cell chronic lymphoblastic leukemia, and several solid tumors (Satterwhite et al., 2001; Yu et al., 2012; Khaled et al., 2015; Zhang et al., 2016; Xutao et al., 2018; Lazarus et al., 2018). However, its role in NKTL development and its clinical value remain unclear. The current study demonstrates that BCL11A expression was upregulated in NKTL. Furthermore, the knockdown of BCL11A induced apoptosis and reduced cell proliferation in NKTL cell lines. Interestingly, the expression of BCL11A may be correlated with those of RUNX3, c-MYC, and P53. Notably, a high BCL11A expression predicted poor outcomes among NKTL patients.

The proto-oncogene BCL11A is located on chromosome 2p16 and is involved in some hematological and solid neoplasms. In B-NHL and HD, BCL11A is involved in tumourigenesis through the gains and amplifications of chromosomes 2p13 and t(2;14) (Satterwhite et al., 2001). Previous studies showed that BCL11A has an oncogenic potential and can promote the development of leukemia in both lymphoid and myeloid lineages (Saiki et al., 2000; Satterwhite et al., 2001). Saiki et al. (2000) demonstrated that BCL11A is downregulated during the myeloid differentiation of HL60 cells, thereby suggesting that BCL11A may cause myeloid leukemia by inhibiting myeloid differentiation. Another study on lymphoid development (Yu et al., 2012) reported that BCL11A is crucial in the lymphopoiesis of B, T, and NK cells. Several studies (Tao et al., 2016; Dong et al., 2017; Xutao et al., 2018; Xu et al., 2018) also revealed an overexpression of BCL11A among patients with acute myeloid and B-cell acute lymphoblastic leukemia, while others (Dong et al., 2017; Xutao et al., 2018) reported that high BCL11A expression predicts worsened prognosis of acute myeloid leukemia.

The clinical value of BCL11A in solid tumors has elicited increasing interest in recent years. Several studies showed that the upregulation of BCL11A contributes to cancer development through various pathways in certain solid tumors. For instance, Khaled et al. (2015) reported that BCL11A is overexpressed in breast cancer and promotes tumor formation. Pathania et al. (2015) found that BCL11A promotes the development of breast cancer by interacting with DNMT1, which is essential for cancer stem cell maintenance and tumourigenesis. Another study on breast cancer (Zhu et al., 2019) revealed that BCL11A enhances the stemness of cancer stem cells and promotes progression by activating Wnt/β-catenin signaling. BCL11A has also been reported to recruit epigenetic complexes by interacting with the histone methyltransferase/deacetylase subunit RBBP4/7 to regulate transcription and promote tumourigenesis in TNBC (Moody et al., 2018). A study on lung squamous carcinoma (LUSC) (Lazarus et al., 2018) reported that BCL11A, as an LUSC oncogene, controls the expression of epigenetic regulators by interacting with SOX2. Another study on lung cancer (Jiang et al., 2013) revealed that BCL11A overexpression is associated with several clinical parameters and is an independent prognostic factor, and is regulated by microRNA-30a and gene amplification.

The results of this study show that BCL11A was overexpressed in NKTL and that the inhibition of BCL11A reduced cell proliferation and increased the apoptosis of NKTL cell lines, thereby suggesting that BCL11A may be an oncogene in NKTL. These results also reveal the essential role of BCL11A in NKTL. The underlying mechanism may be related to the lymphopoiesis of NK/T cells or various oncogenic pathways in solid tumors. Therefore, further studies on this topic are warranted.

To further understand the role of BCL11A overexpression in NKTL, this study examined the correlation of BCL11A expression with RUNX3, c-MYC, and P53 expression levels. Results show that BCL11A expression may be associated with the expression of RUNX3, c-MYC, and P53 in NKTL cells. A recent study on NKTL (Selvarajan et al., 2017) reported an overexpression of RUNX3 in NKTL samples and cell lines. In addition, silencing RUNX3 increases apoptosis and reduces cell proliferation, while MYC upregulates RUNX3 expression in NKTL. Another study (Ng et al., 2011) showed that MYC and P53 are overexpressed in extranodal nasal-type NKTL. These findings are consistent with those of this study. Therefore, the upregulation of BCL11A in NKTL may be correlated with the RUNX3-MYC pathway.

The clinical values of BCL11A expression level in NKTL were also analyzed in this study. NKTL patients were recruited and divided into test and validation groups to examine the prognostic value of BCL11A expression level. The results from the validation group were consistent with those from the test group, thereby enhancing their validity. A high BCL11A expression was shown to be correlated with late-stage and elevated LDH level. The univariate and multivariate analyses identified high BCL11A expression, late stage, and non-CR response as independent unfavorable prognostic factors for OS and PFS among NKTL patients. These findings highlight the functional importance of BCL11A upregulation in NKTL and explain the correlation of high BCL11A with late-stage, elevated LDH levels and worsened prognosis. These findings are also consistent with those of previous reports (Lee et al., 2006; Kim et al., 2016) wherein late-stage, and non-CR response predict worsened OS and PFS in NKTL.

BCL11A was overexpressed in the NKTL cell lines and patient samples, and its inhibition reduced cell proliferation and increased apoptosis. The expression of BCL11A may be correlated with those of RUNX3, c-MYC, and P53. Notably, a high BCL11A expression was correlated with unfavorable clinical features and predicted inferior survival and progression. These results highlight the essential role of BCL11A in the development of NKTL and indicate that its expression may be a promising prognostic biomarker for NKTL. However, this study has several limitations. For instance, while the results reveal that BCL11A was correlated with RUNX3, c-MYC, and P53, such correlation was only tested among a small number of NKTL patients. Nevertheless, these results remain promising despite the small sample. Moreover, the underlying mechanism of such correlation in NKTL remains unclear and warrants further investigation.

## Data Availability Statement

The data that support the findings of this work are available from the corresponding authors upon reasonable request.

## Ethics Statement

All procedures involving human participants were performed in accordance to the ethical standards of the ethics committee and the institutional review board of the Sun Yat-Sen University Cancer Centre and to the 1964 Helsinki declaration and its later amendments or comparable ethical standards. Informed consent was obtained from all participants.

## Author Contributions

HS designed and performed the research. CL analyzed the data and wrote the paper. WF performed the research and analyzed the data. JY, JS, and AP performed the research. HW designed the research and wrote the paper.

## Fnding

This study was funded by the National Natural Science Foundation of China (Contract/Grant Number: 81700148).

## Conflict of Interest

The authors declare that the research was conducted in the absence of any commercial or financial relationships that could be construed as a potential conflict of interest.
