# Supplementary material for: BCL11A Is Oncogenic and Predicts Poor Outcomes in Natural Killer/T-Cell Lymphoma
Source: Front Pharmacol. 2020 Jun 4;11:820. doi: 10.3389/fphar.2020.00820 (PMC7311857; doi:10.3389/fphar.2020.00820)
Supplement: Supplementary file 1 [file DataSheet_1.docx]

**Table S1. Characteristics of NKTL cell lines and culture conditions**.

| **Cell lines** | **Cytokine dependency** | **Culture conditions** |
| --- | --- | --- |
| KHYG1 | IL-2 | RPMI-1640 medium, 10% foetal bovine serum |
| SNK-1 | IL-2 | α-MEM, 20% foetal bovine serum |
| NK-YS | IL-2 | RPMI-1640 medium, 10% foetal bovine serum |
| HANK-1 | IL-2 | α-MEM, 20% foetal bovine serum |

Table S2: List of qPCR primers and siRNA sequences used in this study.

| Gene | Forward Primer (5' - 3') | Reverse Primer (3' - 5') |
| --- | --- | --- |
| BCL11A | CAGCACTTAAGCAAACGGGAAT | TTGTTTCCGTTTGTGCTCGATA |
| GAPDH | ACCCAGAAGACTGTGGATGG | TCTAGACGGCAGGTCAGGTC |
|  | SiRNA Sequence (sense) | |
| BCL11A | GAAUCUACUUAGAAAGCGATT | |
| Control | UUCUCCGAACGUGUCACGUTT | |

Table S3 The clinical characteristics and treatment modalities of validation group of NKTL patients.

| Parameters | Total *n*(%) | BCL11A low expression *n*(%) | BCL11A high expression *n*(%) | *P* value |
| --- | --- | --- | --- | --- |
| Overall | 116(100) | 66(56.9) | 50(43.1) | - |
| Male gender | 75(64.7) | 42(63.6) | 33(66.0) | 0.792 |
| Age >60 years | 21(18.1) | 12(18.2) | 9(18.0) | 0.980 |
| ECOG score ≥2 | 11(9.5) | 4(6.1) | 7(14.0) | 0.148 |
| Ann Arbor stage |  |  |  |  |
| I\II | 90(77.6) | 56(84.8) | 34(68.0) | 0.031 |
| III\IV | 26(22.4) | 10(15.2) | 16(32.0) |  |
| B symptoms | 48(41.4) | 23(34.8) | 25(50.0) | 0.101 |
| LDH>245U/L | 36(31.0) | 15(22.7) | 21(42.0) | 0.026 |
| Chemotherapy regimen |  |  |  |  |
| GELOX | 43(37.1) | 30(45.5) | 13(26.0) | 0.032 |
| EPOCH | 73(62.9) | 36(54.5) | 37(74.0) |  |
| Treatment response |  |  |  |  |
| CR | 52(44.8) | 39(59.1) | 13(26.0) | <0.001 |
| Non-CR | 64(55.2) | 27(40.1) | 37(74.0) |  |

NKTL, natural killer /T-cell lymphoma; BCL11A, B‐cell chronic lymphocytic leukemia/lymphoma 11 A; ECOG, Eastern Cooperative Oncology Group, LDH, lactate dehydrogenase, GELOX, gemcitabine, L-asparaginase, and oxaliplatin; EPOCH, cyclophosphamide, doxorubicin, vincristine, prednisone, and etoposide; CR, complete remission.

Table S4 Univariate and multivariate analyses of prognostic factors in the validation group of NKTL patients.

| Parameters | Overall survival | | | Progression-free survival | | |
| --- | --- | --- | --- | --- | --- | --- |
|  | Univariate analysis | Multivariate analysis | | Univariate analysis | Multivariate analysis | |
|  | P value | HR (95 % CI) | P value | P value | HR (95 % CI) | P value |
| Gender |  |  |  |  |  |  |
| Male vs. female | 0.731 |  |  | 0.625 |  |  |
| Age |  |  |  |  |  |  |
| >60 vs.≤60years | 0.229 |  |  | 0.741 |  |  |
| ECOG scores |  |  |  |  |  |  |
| ≥2 vs. 0-1 | 0,368 |  |  | 0.242 |  |  |
| Stage |  |  |  |  |  |  |
| III\IV vs. I\II | <0.001 | 2.00(1.03-2.91) | 0.042 | <0.001 | 2.06(1.07-3.97) | 0.030 |
| B symptoms |  |  |  |  |  |  |
| Yes vs. no | 0.036 |  |  | 0.025 |  |  |
| LDH |  |  |  |  |  |  |
| Elevated vs. normal | 0.001 |  |  | 0.002 |  |  |
| Chemotherapy |  |  |  |  |  |  |
| GELOX vs. EPOCH | 0.006 |  |  | 0.013 |  |  |
| Treatment response |  |  |  |  |  |  |
| Non-CR vs. CR | <0.001 | 5.18(1.88-14.31) | 0.001 | <0.001 | 2.29(1.09-4.82) | 0.029 |
| BCL11A expression |  |  |  |  |  |  |
| High vs. low | <0.001 | 3.22(1.58-6.58) | 0.001 | <0.001 | 3.98(2.08-7.58) | <0.001 |

NKTL, natural killer /T-cell lymphoma; HR, hazard ratio; CI, confidence interval; ECOG, Eastern Cooperative Oncology Group; LDH, lactate dehydrogenase; GELOX, gemcitabine, L-asparaginase, and oxaliplatin; EPOCH, cyclophosphamide, doxorubicin, vincristine, prednisone, and etoposide; CR, complete remission; BCL11A, B‐cell chronic lymphocytic leukemia/lymphoma 11 A.
